# The Creative Stereotype Effect

**DOI:** 10.1371/journal.pone.0142567

**Published:** 2016-02-10

**Authors:** Denis Dumas, Kevin N. Dunbar

**Affiliations:** Department of Human Development and Quantitative Methodology, University of Maryland, College Park, Maryland, United States of America; University of Georgia, UNITED STATES

## Abstract

Because of its fundamental relevance to scientific innovation, artistic expression, and human ingenuity, creativity has long been the subject of systematic psychological investigation. Concomitantly, the far-reaching effects of stereotypes on various cognitive and social processes have been widely researched. Bridging these two literatures, we show in a series of two studies that stereotypes related to creativity can both enhance and diminish individuals’ performance on a divergent thinking task. Specifically, Study 1 demonstrated that participants asked to take on a stereotypically uninhibited perspective performed significantly better on a divergent thinking task than those participants who took on a stereotypically inhibited perspective, and a control group. Relatedly, Study 2 showed that the same effect is found within-subjects, with divergent thinking significantly improving when participants invoke an uninhibited stereotype. Moreover, we demonstrate the efficacy of Latent Semantic Analysis as an objective measure of the originality of ideas, and discuss implications of our findings for the nature of creativity. Namely, that creativity may not be best described as a stable individual trait, but as a malleable product of context and perspective.

## The Creative Stereotype Effect

From Wall Street to Silicon Valley and even your local kindergarten; a multi-million-dollar industry has emerged that attempts to identify and cultivate the next generation of creative people. However, despite the long-standing search for the essence of creativity, it has remained shrouded in stereotypes of creative geniuses such as Leonardo Da Vinci, Ai Wei Wei, and Steve Jobs. Nowhere has this quest for creativity been more evident than in the development of tests of creativity. Many researchers and the general public have assumed that being creative is largely a trait that we are born with (e.g., [1, 2]). Here, we take a very different approach and demonstrate that the ways in which we instantiate stereotypes of creativity can either increase or decrease our creative performance, a phenomenon we label the Creative Stereotype Effect.

When the Soviet Union appeared to be winning the space race in the 1950s and 1960s, the search for creative people began in earnest. Psychologists such as Guilford [3,4], Hudson [5] and Torrance [6] developed tests to identify creative individuals. Today, the economic, political, and cultural problems of the world have made the search for creativity no less relevant and have led to a renewed call for the fostering of creative thought. As such, a new generation of creativity researchers in a wide variety of disciplines including psychometrics (e.g., [6]), neuroscience (e.g., [7, 8]), and social psychology (e.g., [9]) have refined our understanding of creativity and the emphasis has shifted from seeing creativity as an inherent ability to discovering ways of making people more creative.

Methods of stimulating creativity in Science, Engineering, Business, the Arts, and the Humanities, have leaped to the forefront of political, economic, and research agendas across the world (e.g., [10,11]). In general, the effectiveness of these methods of stimulating creativity stem from tasks that remove self or socially imposed constraints on creative thinking [12]. In this investigation we conceptualize a previously unidentified constraint on creativity—stereotypes—and use stereotypes to both enhance and attenuate individuals’ performance on a divergent thinking task that is frequently used to assess creative potential.

## Components of Creativity

Divergent thinking tasks, which require people to generate as many original ideas as possible, have been among the most common measures used in the creativity literature for decades [6,13,14,15,16]. The ability to engage in divergent thinking is essential to creativity as it allows people to see problems in multiple ways, generate novel solutions, concepts, and ideas. As such, performance on divergent thinking tasks is thought to be an accurate measure of creative potential [13,17]. Performance on divergent thinking tasks is typically conceptualized in terms of multiple factors, and hence a number of methods for assessing creative potential in divergent thinking have been developed [18].

One frequently used procedure for scoring divergent thinking tasks is the number of ideas generated, known as fluency [4,14,16]. Fluency can be calculated quickly, objectively, and easily by counting the number of ideas generated. However, while fluency has demonstrated efficacy in testing hypotheses in the field of creativity, fluency only accounts for the quantity of ideas and not for the originality of those ideas. In contrast to fluency, originality explicitly takes into account the creative quality of an idea by assessing the degree to which that idea was novel. As such, studies that utilize fluency as a scoring method for divergent thinking tasks frequently also find that measuring originality necessary to tap creative potential [16]. This is because, while all accepted definitions of creativity include the originality of ideas as central to creativity, fluency is not considered central to the definition of creativity and may more appropriately be described as lying to the periphery of the construct itself [19].

Assessing originality has been implemented in a variety of ways [6, 7, 13, 20, 21]. One modern approach has been to assess the semantic distance of participant responses from the original prompt [13, 22, 23, 24, 25]. One measure of semantic distance that has proved effective for scoring the originality of ideas is Latent Semantic Analysis (LSA) [24, 25, 26, 27]. LSA is a computational method that uses a very large body of text, called a corpus, to quantify the semantic relation between and among terms [28]. LSA has been shown to account for performance on a wide variety of tasks that assess how semantically distant two concepts are away from each other. For example; word priming [29], category membership [30], and essay scoring [31]. LSA calculates originality as the semantic distance between a generated idea and its corresponding prompt or starting point [26]. LSA produces a measure of originality based on the semantic space of the language (e.g., English) in which divergent thinking was undertaken. Because LSA is objective, captures the distance between two ideas, and reveals how often two ideas are mentioned together, we use LSA as a measure of Originality.

Recently, a number of studies have demonstrated that creative ability—either in terms of fluency, originality, or both can be improved by a wide variety of methods. For example, divergent thinking can be altered through meditation [32], diet [10], walking [11], music [33], cannabis [34], or mood [9]. Indeed, the literature demonstrating the malleability of divergent thinking is becoming quite robust, with the positive influence of traveling for vacation [35] and multicultural experiences [36] also being empirically identified. Thus, there is a growing body of evidence that divergent thinking is indeed malleable.

Importantly, this body of work stands in stark contrast to arguments that conceptualize creativity as a relatively stable individual trait (e.g., [37]). Indeed, as a set, the more contextually manipulated investigations have suggested that creativity instead may be a more malleable state than previously thought. Interestingly, these types of results are consistent with hypothesized mechanisms that operate not by directly enhancing a participant’s creative ability, but by removing constraints on creativity (e.g., inhibition; [12, 38]. It can be hypothesized that the removal of constraints on creativity, whether they be contextual, cognitive, or social, may be effective ways of enhancing divergent thinking. However, the nature of the manipulations used in previous studies of creative potential may be a limiting factor, in that many manipulations (e.g., meditation, cannabis) are difficult to control, time consuming, and potentially so diverse that the underlying mechanisms enhancing divergent thinking are difficult to discern. In order to maximize the usability of such manipulations, more efficient, generalizable, and reliable manipulations must be developed. Below we outline one such manipulation.

## Stereotypes and Creativity

Awareness of salient stereotypes associated with better or worse performance on a given task has been repeatedly shown to affect a wide range of cognitive and social processes [39]. For instance, if an individual believes that a social group they belong to should perform poorly on a particular task, their performance will be attenuated [40]. Stereotypes can also produce better performance if the individual believes their group should or will perform well on a given task [41]. In this way, stereotypes can both enhance the performance of those who instantiate a positive stereotype, and lower the performance for those who instantiate a negative stereotype. Similarly, stereotypes allow individuals to make positive and negative inferences about particular occupations, regardless of whether or not they identify with a given occupation or stereotype (e.g., [42]). Here, we manipulate the use of occupational stereotypes to enhance or diminish divergent thinking.

Interestingly, even before the now well-known effects of stereotypes were documented, Liam Hudson [5] made an early attempt to use stereotypes to improve the creative potential of elementary-school-aged students. Hudson showed that students who were asked to think of themselves as diligent scientists performed significantly worse on a divergent thinking task than students asked to think of themselves as eccentric artists. From this work, evidence emerged in that directly asking participants to be creative is effective (e.g., [43]). However, to our knowledge, the effect that Hudson first documented has never been conceptualized as asking participants to use stereotypes while performing a test of creative potential. We propose that our method could be a constraint-removing activity that can lead to improved performance on a divergent thinking task. In two studies, we investigated whether divergent thinking can be enhanced or diminished by invoking stereotypes of highly creative or less creative occupations while performing the Uses of Objects task.

## Study 1

### Method

#### Participants

Ninety six undergraduate students at a large mid-Atlantic university (59 female; 61.45%) participated in this study. Students had a declared major in Biology (n = 24; 25.00%), Physics (n = 24; 25.00%), Art (n = 24; 25.00%), or Theater (n = 24; 25.00%). Participants were recruited via posters displayed around the university campus, or digital postings to university listservs. In exchange for their participation, participants were entered into a lottery where they could win an iPad. Participants ranged in age from 18 to 23 years old, with a mean age of 20.00 years old (*SD* = 1.54). The sample was diverse with 58.33% of students reporting their ethnicity as White (n = 56); 8.33% African American (n = 8); 15.63% Hispanic/Latino (n = 15); and 17.71% Asian (n = 17). Participants reported a mean grade point average of 3.43 (*SD* = .54) on a four point scale, with GPAs ranging from 2.00 to 4.00.

#### Measures

The Uses of Objects Task (UOT), a psychometric test that requires participants to generate multiple original uses for a given object, was used. The UOT has been widely used in research on creativity for many years [4, 5, 44, 45]. The UOT was administered online, using the Qualtrics online service platform. Online participation outside of the laboratory was considered advantageous because participants could complete the UOT from any computer connected to the Internet, allowing a degree of privacy and flexibility.

The names of ten different objects were presented to participants in a random order on a computer screen. The ten included objects were chosen based on an empirical norming study of students at the university where this research took place [46]. The object-names that were presented were: book, fork, table, hammer, pants, trumpet, truck, carrot, shovel, and sandals. Based on empirical norms [46] each of these objects was of medium-level typicality [47] for their respective categories. Participants were given two minutes to provide uses for each object before they were automatically advanced to the next object in the task.

#### Procedure

After receiving a link to the study website, participants completed an informed consent form. It should be noted that the University of Maryland, College Park Institutional Review Board specifically approved this research, including both studies described in this paper. For this research, written consent was collected from participants via a consent form. Our university's IRB approved the consent form itself, as well as the consent procedure. Explicitly on our consent form, a student's signature indicated that they were "at least 18 years of age; you have read this consent form or have had it read to you; your questions have been answered to your satisfaction and you voluntarily agree to participate in this research study."

Then, because they were participating from a computer outside the laboratory, participants were asked to minimize the distractors around them. Specifically, they were asked to turn off music, close other websites, and/or turn off the TV. Further, because participation required a significant amount of typing, for which a traditional keyboard may have been important, participants were asked not to participate on a smartphone or tablet. Then, participants were given general task instructions and were randomly given one of two stereotypes for the UOT, or were placed in the control condition.

#### Stereotypes

Pilot testing revealed two stereotypes related to creativity that were highly salient to our target population of undergraduate students: the eccentric poet and the rigid librarian. Pilot tests indicated that undergraduates generally regarded poets as a type of person who is creative, uninhibited, and eccentric, and librarians as a type of person who is uncreative, rigid or inflexible. Of course, we as researchers, do not believe that librarians are, in reality, rigid and uncreative. However, this stereotype, which seems to be generally held by undergraduate students—so much so that a quick Google search reveals multiple websites and blogs produced by librarians dedicated to dispelling it—was precisely the type of stereotype we wanted to draw on. The framings of the stereotype that students received for the eccentric poet or the rigid librarian conditions were as follows:

As you complete the Uses of Objects Task, please imagine that you are an eccentric poet.

As you complete the Uses of Objects Task, please imagine that you are a rigid librarian.

A control condition was also included, in which participants did not receive a particular stereotype, but only received general task instructions. Randomization settings on the online platform insured that equal numbers of students with each declared major (i.e., biology, physics, art, and theater) were randomly placed into each of the three conditions (i.e., eccentric poet, rigid librarian, and control). Participants completed all ten randomly ordered objects under the same condition. Participants supplied demographic information after completing the UOT.

### Results

#### Scoring of the UOT

The UOT was scored in two different ways, producing two different outcome variables for analysis. The first scoring method was determining fluency by counting the number of uses that the participants produced for each object, the second was scoring originality through Latent Semantic Analysis (LSA). Each of these scoring methods will now be further explicated.

#### Fluency

The uses provided by each participant for each use were counted. Then, the counts associated with each of the ten objects were summed to create a composite score for each participant. This “total uses” outcome variables represents a participants’ creative fluency. The mean number of uses produced across all ten objects in our sample was 77.79 (*SD* = 33.91).

#### Originality

LSA is a statistical technique for extracting and representing the similarity of word meanings through the analysis of a large body of text, called a corpus [28]. Specifically, the frequency of each word within a body of text is represented in matrix form, then, after undergoing a statistical dimensionality reduction, the latent relations between word-vectors are calculated by taking the cosine of the angle between word vectors [48]. Importantly, latent variable correlations, such as those derived in factor analysis, can always be calculated in this way, by taking the cosine of the angle between variable vectors in multivariate space. Interestingly, LSA has been shown to be more reliable than human coders at scoring the originality of ideas [24, 25].

Semantic similarity measures were calculated using 300 factors (the most typical number used in LSA), with a very large (i.e., 37,651 included documents and more than 11 million words) English language corpus built to approximate the expected reading experience of an average college student (*general-reading-up-to-the-first-year-in-college)* [29]. A semantic similarity measure ranging between -1 and 1 was produced for each use provided. In order to create a readily interpretable measure of semantic distance, semantic similarity scores were subtracted from 1, resulting in a semantic distance measure that ranged from 0 as a minimum to 2 as a maximum. Then, in order to produce a measure of originality that was relatively independent from the total uses (i.e., fluency) outcome variable, the mean of the semantic distance scores associated with each of the ten objects on the UOT was calculated, then summed, to produce a composite semantic distance (i.e., originality) outcome variable, which represents a participant’s originality. The mean semantic distance score in our sample across all ten objects was 7.61 (*SD* = .69). The two outcome variables (i.e., fluency and originality) were correlated at *r* = .36 (*p* < .001), implying that, while they are positively related, they contribute much non-overlapping information about participants divergent thinking ability.

#### Stereotype manipulation

To examine the effect of the framing conditions on participants’ divergent thinking, mean differences on our two outcome variables were compared. All group means and corresponding standard deviations for this study are presented in Table 1. We first used ANOVAs to test for significant mean differences on either of the outcome variables among students with declared majors in biology, physics, art, or theater. No significant differences were found on either the fluency [*F*(3,93) = .95, *p* = .42, *η*_p_^2^ = .03] or originality [*F*(3,93) = .13, *p* = .94, *η*_p_^2^ = .004] of the uses produced among the four majors. Therefore, we combined the sample for the subsequent analysis.

**Table 1:** Outcome variable means by major and stereotype condition for Study 1.

Major	Condition	N	Total Uses	Semantic Distance
Full Sample	All Conditions	96	*M* = 77.79, *SD* = 33.91	*M* = 7.61, *SD* = .69
	Rigid Librarian	32	*M* = 60.34, *SD* = 24.38	*M* = 7.16, *SD* = .78
	Control	32	*M* = 77.88, *SD* = 32.20	*M* = 7.63, *SD* = .57
	Eccentric Poet	32	*M* = 92.16, *SD* = 36.99	*M* = 8.03, *SD* = .39
Biology	All Conditions	24	*M* = 69.97, *SD* = 24.24	*M* = 7.53, *SD* = .84
	Rigid Librarian	8	*M* = 56.75, *SD* = 21.42	*M* = 6.88, *SD* = 1.02
	Control	8	*M* = 74.25, *SD* = 22.65	*M* = 7.67, *SD* = .61
	Eccentric Poet	8	*M* = 78.38, *SD* = 25.65	*M* = 8.04, *SD* = .36
Physics	All Conditions	24	*M* = 73.96, *SD* = 39.76	*M* = 7.63, *SD* = .75
	Rigid Librarian	8	*M* = 61.38, *SD* = 25.02	*M* = 6.97, *SD* = .87
	Control	8	*M* = 63.25, *SD* = 16.51	*M* = 7.83, *SD* = .42
	Eccentric Poet	8	*M* = 97.25, *SD* = 58.00	*M* = 8.12, *SD* = .37
Art	All Conditions	24	*M* = 77.71, *SD* = 30.55	*M* = 7.65, *SD* = .62
	Rigid Librarian	8	*M* = 64.38, *SD* = 24.47	*M* = 7.40, *SD* = .61
	Control	8	*M* = 81.63, *SD* = 32.66	*M* = 7.55, *SD* = .74
	Eccentric Poet	8	*M* = 87.13, *SD* = 32.85	*M* = 8.00, *SD* = .37
Theater	All Conditions	24	*M* = 85.71, *SD* = 38.78	*M* = 7.62, *SD* = .57
	Rigid Librarian	8	*M* = 58.88, *SD* = 30.197	*M* = 7.40, *SD* = .53
	Control	8	*M* = 92.38, *SD* = 47.15	*M* = 7.47, *SD* = .52
	Eccentric Poet	8	*M* = 105.88, *SD* = 21.65	*M* = 7.99, *SD* = .53

**Fig 1:**
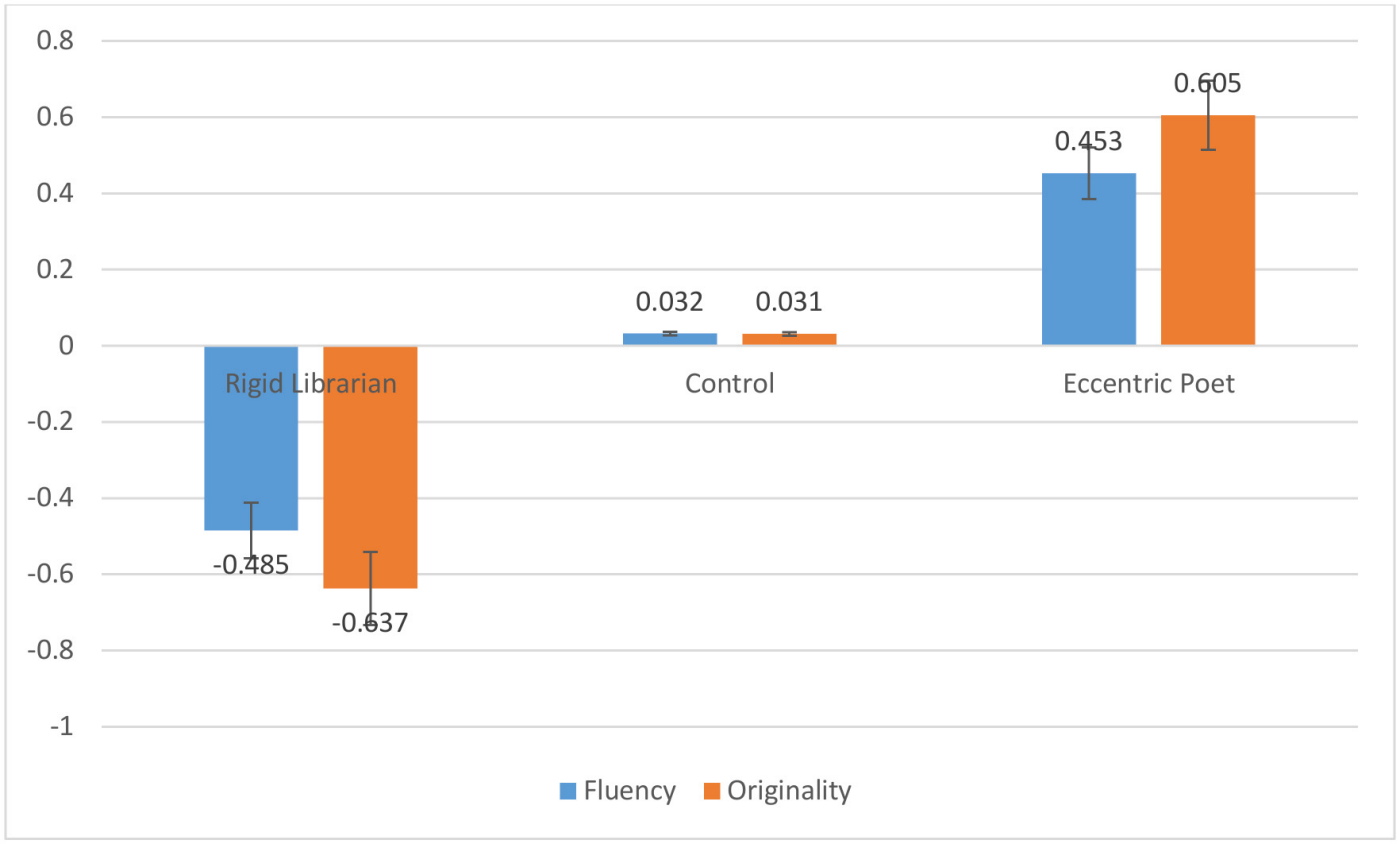
Standardized fluency and originality scores in each of three conditions for Study 1.

**Fig 2:**
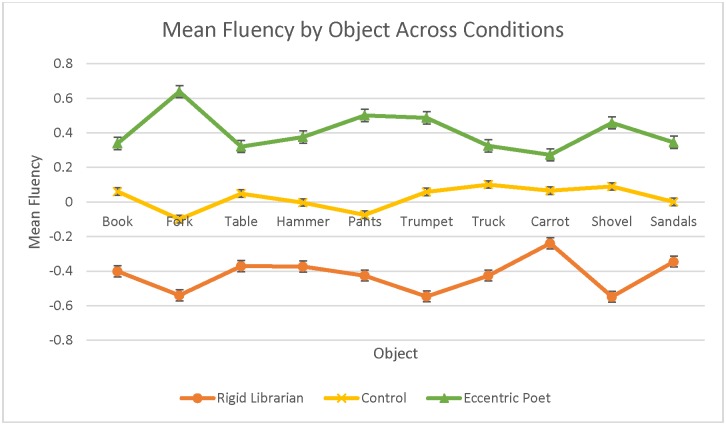
Standardized fluency scores by item for Study 1.

**Fig 3:**
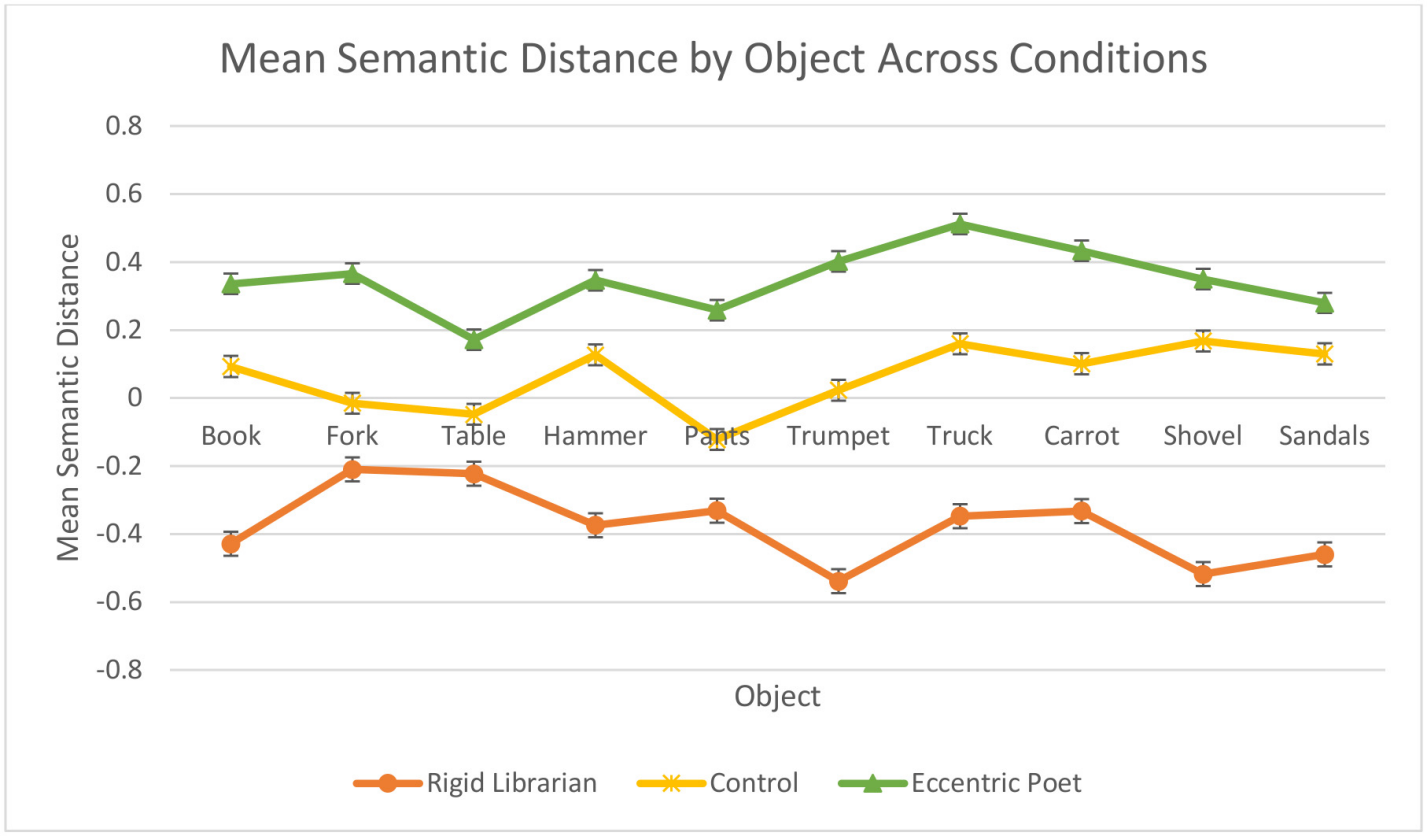
Standardized originality scores by item for Study 2.

We found that significant mean differences in fluency [*F*(2,93) = 8.12, *p <* .001, *η*_p_^2^ = .15] and originality [*F*(2,93) = 16.40, *p <* .001, *η*_p_^2^ = .26] existed between the three framing conditions, with participants in the rigid librarian condition scoring lowest, those in the control condition scoring in the middle, and those in the eccentric poet condition scoring highest on both outcome variables. Moreover, pairwise comparisons using the Scheffé procedure were used to identify which particular differences in the outcome variables across the groups were significant for this sample. Specifically, the mean difference in fluency between the eccentric poet and rigid librarian stereotypes was significant [*F*(2,93) = 31.81, *p <* .001], while the difference between the control group and each of the framing conditions was not significant in terms of fluency. However, in the analysis pertaining to originality, each of the pairwise comparisons were significant: rigid librarian to control [*F*(2,93) = .467, *p* = .011], rigid librarian to eccentric poet [*F*(2,93) = .869, *p <* .001], and control to eccentric poet [*F*(2,93) = .401, *p* = .034]. In order to support visual interpretation of these results, group means (i.e., poet, librarian, and control) on each of the outcome variables (i.e., fluency and originality) were standardized, so as to represent the difference in standard deviations of that group’s mean from the grand-mean of the entire sample. Then, these standardized values were plotted in Fig 1. As can be seen from this Fig, the stereotype both negatively and positively affected the divergent thinking of participants in the predicted directions. Moreover, as Figs 2 and 3 illustrate, this effect was obtained across all items in the UOT, both in terms of fluency (Fig 2) and originality (Fig 3), demonstrating the consistency of the creative stereotype effect.

## Study 2

The results of Study 1 demonstrated the power of stereotypes to alter divergent thinking across participants and in comparison to a control group. In Study 2, we go one step further and ask whether the same participant can produce more divergent responses in one stereotype framing context and less divergent responses in a different stereotype framing context.

### Method

#### Participants

One hundred and five undergraduate students at a large mid-Atlantic university (72 female; 68.57%) participated in this study. Because major was not a significant factor in Study 1, major was held constant in Study 2. Therefore, participants were recruited through a university online research participation portal, in which Psychology students are required to enroll. In exchange for their participation, students received research participation credit. Participants ranged in age from 17 to 27 years old, with a mean age of 19.54 years old (*SD* = 1.53). The sample was highly diverse, with 60.00% of students reporting their ethnicity as White (n = 63); 4.76% African American/Black (n = 5); 9.52% Hispanic/Latino (n = 10); and 28.57% Asian (n = 30). Participants reported a mean grade point average of 3.42 (*SD* = .46) on a four point scale, with GPAs ranging from 2 to 4.

#### Procedure

The procedure for study 2 was similar to study 1, with participants receiving the same general instructions for the UOT task. Further, the same two stereotypes (i.e., eccentric poet and rigid librarian) were used. Most importantly, Study 2 utilized a within-subjects design, in which participants completed the first half of the UOT (5 objects), in an initial randomized stereotype condition (poet or librarian), which took 10 minutes. As with Study 1, participants were given 2 minutes to generate uses for each object before they were automatically advanced to the next object in the task. After the first 5 objects, the participants were notified that they had completed the first part of the study, and that they were going be given new directions for the UOT. Then, they were presented with the stereotype (either poet or librarian) that they had *not* been randomly assigned at the beginning of the study, with the order of the presentation counterbalanced so that equal numbers of participants received the poet and librarian frame first. After receiving their second stereotype, the participants completed the remaining 5 randomized objects, which took another ten minutes. Finally, participants provided demographic information and logged out of the study website.

### Results

#### Scoring of the UOT

As with study 1, the UOT was scored using counts and LSA, producing two outcome variables for analysis: total uses, and semantic distance. The mean number of uses produced across all ten objects was 62.43 (*SD* = 29.77), and the average semantic distance across all ten objects was 7.19 (*SD* = .82). The observed correlation between the two outcome variables was *r* = .20 (*p* = .043).

#### Stereotype manipulation

**Fig 4:**
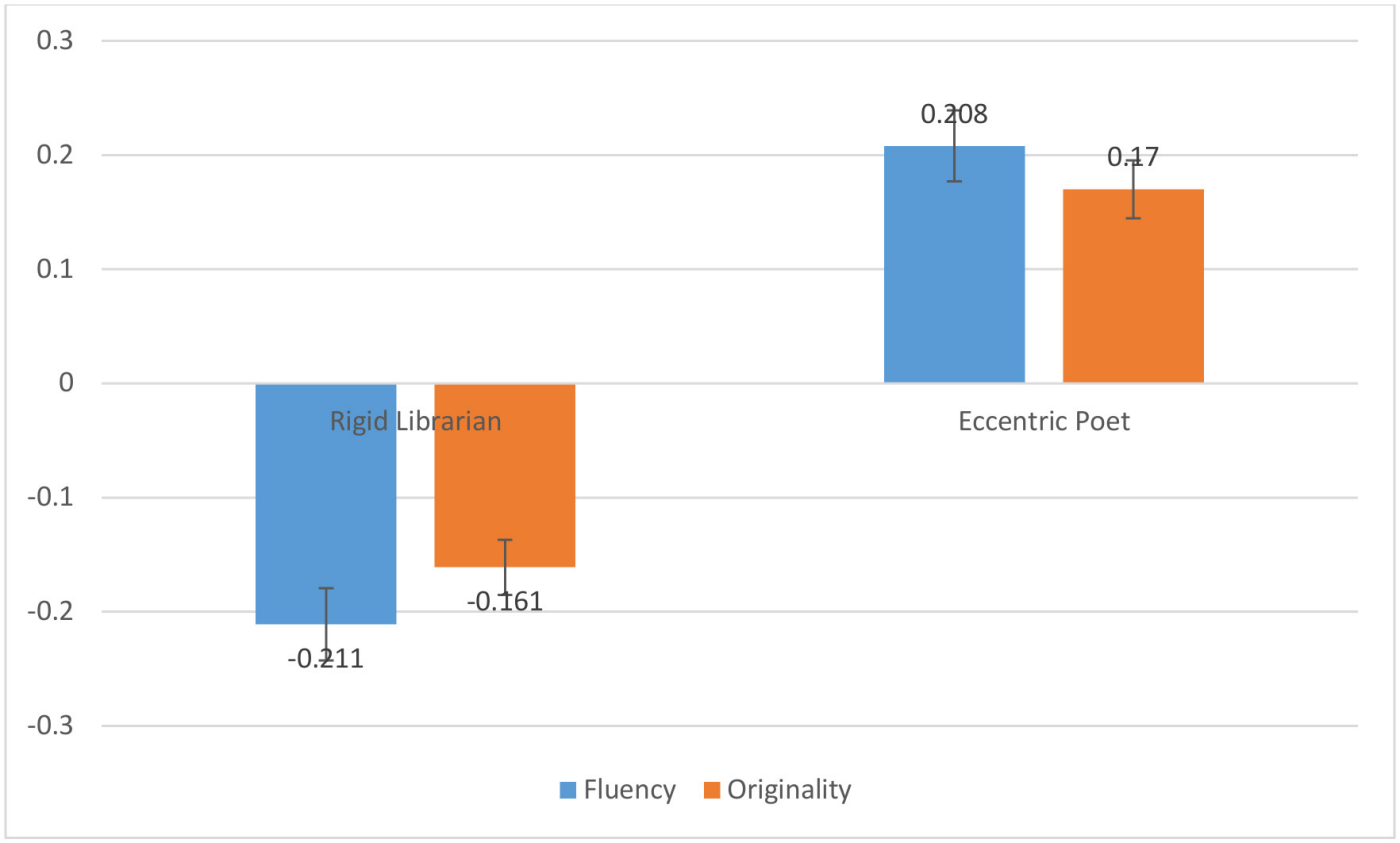
Standardized fluency and originality scores in each of two conditions for Study 2.

In order to examine the effect of the stereotype manipulation on participants’ divergent thinking, the means of both our outcome variables (i.e., fluency and originality) were compared across conditions. The mean number of uses produced by participants for the five objects they encountered in the eccentric poet condition was 34.90 (*SD* = 19.91), while in the rigid librarian condition, 27.52 (*SD* = 14.33) uses were produced on average. A paired-sample t-test [*t* = 4.25 (*df* = 104), *p* < .01, *d* = .84], was used to confirm that the mean difference between the framing conditions was statistically significant, and that the effect of the framing condition was large. The mean semantic distance of the uses provided by participants was 3.67 (*SD* = .44) while in the eccentric poet condition and 3.52 (*SD* = .51) while in the poet condition. As with fluency, the shift in originality (semantic distance) was significant and the effect size was relatively large [*t* = 3.44(*df* = 104), *p* < .01, *d* = .67]. As with study 1, condition means were standardized, representing the difference in standard deviations of the mean of the specific condition (i.e., poet or librarian) from the grand-mean including both conditions. These standardized values were plotted in Fig 4.

## Conclusion

Across two studies, we have shown that divergent thinking, a key indicator of creative potential, can be significantly attenuated or enhanced depending on the type of stereotype invoked while performing the task. Furthermore, as can be seen in Figs 2 and 3, we have demonstrated that the effect occurs irrespective of the item on the UOT. To our knowledge, these studies are the first to use stereotypes to both diminish and improve divergent thinking performance both within and across individuals. Three principal implications can be drawn from these studies: (a) that divergent thinking, including both fluency and originality, is highly malleable rather than a fixed trait, (b) that stereotypes can be used to diminish or enhance creative output from diverse groups of participants, and (c) that stereotype manipulation is an effective way of altering divergent thinking that can be applied across different contexts.

Recently, some researchers (e.g., [11]) have demonstrated that divergent thinking may not be best described as a stable individual difference trait, but rather as a malleable product of context and perspective. However, despite these types of demonstrations, some researchers and the general public continue to implicitly or explicitly describe creativity as a stable trait (e.g., [2, 37]). Conversely, in this investigation, divergent thinking was significantly reduced or enhanced depending upon which stereotypical perspective participants took. The finding that divergent thinking may not be stable, but is instead highly influenced by contextual factors such as stereotypes may be a new and especially useful tool for enhancing creative performance. Moreover, we found that when participants took on an uninhibited stereotype (i.e., eccentric poet) *both* their fluency and originality was significantly enhanced.

Stereotypes previously have been identified as capable of affecting a wide variety of cognitive and social processes (e.g., [39, 41]). However, this is the first investigation of which we are aware to demonstrate that stereotypes of creativity can both inhibit or enhance divergent thinking, depending on the context. Importantly, unlike much of the stereotype-threat literature, these studies did not utilize stereotypes related to ethnicity or gender, but instead included stereotypes based more generally on profession or personal interest. Moreover, in contrast to the stereotype-threat literature, participants in this study were not meant to personally identify with a given stereotype, but rather their performance may have been enhanced or diminished through a process of stereotype activation or priming. Going forward, identifying salient stereotypes that can inhibit or enhance creativity or other cognitive processes may be of particular importance, especially in domains where improving the creative performance of individuals is an important goal.

In addition to demonstrating that stereotypes can be used to enhance or diminish divergent thinking, our initial experiments reported here raise a number of questions regarding the role of different factors influencing the Creative Stereotype Effect. One question is whether the amount of time given to participants to respond to a divergent thinking task influences the strength or valence of the effect. For example, longer time-limits or no time-limit at all to participant responses may produce an even larger effect, or alter the way the effect occurs.

Another question which our research raises is whether the ways that stereotypes are presented to participants influences the effect. In the two experiments presented here, adjectives (i.e. (eccentric and rigid) and occupations (i.e., poet and librarian) were coupled in such a way to reinforce the existing stereotype. However, whether or how the Creative Stereotype Effect might change when the adjective does not stereotypically match the occupation, or whether only one or another of these terms is sufficient to create the effect, remains an empirical question for future work.

Recently reports of a creativity crisis have emerged in both the popular press and research reports; in these reports it has been argued that divergent thinking and creative ability have been steadily declining over the past two decades (e.g., [49, 50]). This observation implies that rather than fostering creativity, educational and economic systems appear to be decreasing it. However, based on the findings of the current investigation, one potential explanation for the observed declines in divergent thinking is the perspective from which test takers approach the task. In any system that places an emphasis on test-scores, test takers may feel compelled to adopt a rigid perspective when performing a creative task. Therefore creativity may not be declining, rather test takers may be adopting a more and rigid perspective or stereotype, hindering their performance. Therefore, it is important to determine the stereotypes that individuals invoke when their divergent thinking or creativity is being assessed. As the results of these studies show, provision of different stereotypes is an effective approach to enhancing divergent thinking: a key indicator of creative thought.

## Supporting Information

S1 Data The minimal dataset related to this investigation is uploaded as supplemental information. (XLSX)
